# Neonatal Gonococcal Conjunctivitis Caused by Fluoroquinolone-Resistant *Neisseria gonorrhoeae*

**DOI:** 10.3201/eid3110.250895

**Published:** 2025-10

**Authors:** Hiroto Mizushima, Miwa Komori, Carolina Andrea Yoshida, Isao Miyairi

**Affiliations:** Hamamatsu University School of Medicine, Shizuoka, Japan

**Keywords:** neonatal ophthalmia, bacteria, bacterial infections, conjunctivitis, fluoroquinolone, prophylaxis, *Neisseria gonorrhoeae*, *Chlamydia trachomatis*, Japan, antimicrobial resistance

## Abstract

Prophylaxis for ophthalmia neonatorum remains in use despite decreased incidence of the condition. We report a breakthrough case of neonatal conjunctivitis in Japan caused by a levofloxacin-resistant *Neisseria gonorrhoeae* bacteria strain, co-infected with *Chlamydia trachomatis* bacteria. This case highlights failures in screening, prophylaxis, and treatment, underscoring the need to reassess prevention strategies.

Since the introduction of prophylactic ophthalmic solutions by Carl Credé in 1881, the incidence of neonatal gonococcal conjunctivitis has declined markedly (*1*,*2*). Over time, the agents used for ocular prophylaxis have shifted from silver nitrate to erythromycin or tetracycline ophthalmic ointments and povidone/iodine (*3*).

In Japan, erythromycin/colistin ophthalmic formulations had been widely used since 1970. However, production of erythromycin/colistin preparations was discontinued in 2015, prompting some institutions to adopt fluoroquinolone-based ophthalmic agents for neonatal prophylaxis. Although fluoroquinolone-resistant *Neisseria gonorrhoeae* bacteria strains have been reported in adults, neonatal infections with such strains remain rare (*2*). We describe a breakthrough case of neonatal gonococcal conjunctivitis caused by a fluoroquinolone-resistant *N. gonorrhoeae* strain and further complicated by concurrent *Chlamydia trachomatis* bacteria infection. This case highlights the need to reevaluate current strategies for preventing and managing neonatal conjunctivitis.

In 2023, a 12-day-old female infant was brought to a hospital in Shizuoka, Japan, where she was observed to have purulent ocular discharge and periorbital swelling. She was born at full-term through spontaneous vaginal delivery. The mother had a negative *C. trachomatis* nucleic acid amplification test at 12 weeks’ gestation; however, no screening for *N. gonorrhoeae* was performed. The infant received prophylactic levofloxacin ophthalmic solution immediately after birth. From day 4 of life, purulent ocular discharge started occurring and progressively worsened, despite use of levofloxacin ophthalmic solution, applied 3 times daily, that was prescribed by a local pediatrician on day 8. By day 10, bilateral eyelid edema and marked periorbital inflammation were evident, prompting referral to the hospital’s ophthalmology department.

We obtained conjunctival cultures and added a cephalosporin-based ophthalmic preparation to the patient’s treatment plan. Culture of the ocular discharge confirmed *N. gonorrhoeae* infection, leading to a diagnosis of neonatal gonococcal conjunctivitis. We then admitted the infant for treatment.

At admission, we recorded vital signs, laboratory test results, and urine test results (Appendix). Physical examination revealed bilateral conjunctival injection, pronounced left eyelid edema, and periorbital erythema (Figure). The corneas were clear, having no evidence of epithelial defect, ulceration, or perforation. We noted no anterior chamber abnormalities, and ocular motility was preserved. We observed a conjunctival hemorrhage in the left eye.

**Figure:**
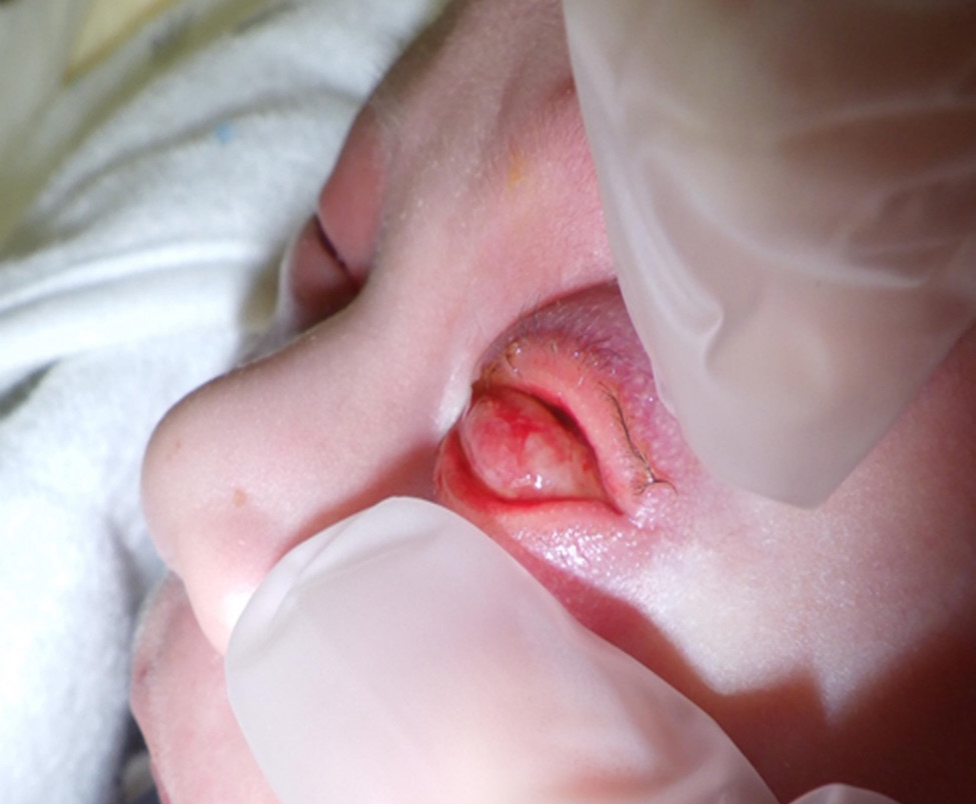
Bilateral conjunctival infection, pronounced left eyelid edema, and periorbital erythema observed in a 12-day-old infant in case of neonatal gonococcal conjunctivitis caused by fluoroquinolone-resistant *Neisseria gonorrhoeae*, Japan, 2023.

We initiated intravenous cefotaxime after hospitalization. We discontinued antimicrobial therapy after 48 hours of negative blood culture results. Ocular symptoms began improving by hospital day 3.

A PCR test performed on a pharyngeal swab sample on hospital day 3 was positive for *C. trachomatis*, and we administered azithromycin. The infant recovered fully without recurrence.

The *N. gonorrhoeae* isolate, which we designated as B196-JP22, was resistant to levofloxacin (MIC 12 μg/mL), had elevated MIC for azithromycin (MIC 0.75 μg/mL), and had reduced susceptibility to penicillin G (1 μg/mL) (Table). We performed full-genome analysis on the isolate (Appendix) and deposited it to the National Center for Biotechnology Information BioProject database (project no. PRJNA1277472) and BioSample database (accession no. SAMN49109531). We found missense mutations in the *gyrA* and *parC* genes consistent with the levofloxacin-resistance phenotype (*5*) and antimicrobial resistance genes (Table) (Appendix).

**Table:** MICs of antimicrobial drugs for *Neisseria gonorrhoeae* isolate B196-JP22 from a case of neonatal gonococcal conjunctivitis caused by fluoroquinolone-resistant *N. gonorrhoeae*, Japan

Antimicrobial drug	MIC, mg/L	Interpretation*	Antimicrobial resistance genes (mutation details)
Penicillin G	1	Reduced susceptibility	*mtrR* (mtrR_A39T),† *mtrR* (mtrR_promoter_a-57del),‡ *ponA* (ponA_L421P),† *penA* gene§
Piperacillin/tazobactam	<0.01	Susceptible	
Ceftriaxone	0.05	Susceptible	
Meropenem	0.01	Susceptible	
Levofloxacin	12	Resistant	*gyrA* (gyrA_S91F/D95N),† *parC* (parC_S87I)†
Azithromycin	0.75	Elevated MIC¶	*mtrA*, *macA*, *macB*, *farA*#

*Antimicrobial MIC tested by using Etest (bioMérieux, https://www.biomerieux.com). Interpretation according to Clinical and Laboratory Standards Institute (CLSI) standards (*4*). CLSI cutoff for susceptible is MIC *<*0.06 μg/mL, for intermediate is MIC 0.12–1 μg/mL, for resistant is MIC >2 μg/mL. A MIC of 1 was described as reduced susceptibility. †Missense mutation. ‡Promoter deletion. §The *penA* gene that encodes the PBP2 shared 99.77% identity with the *penA* encoded by the World Health Organization Alfa strain. However, it could not be classified by using the NG-STAR platform (https://ngstar.canada.ca) because of 2 gaps in the sequence, and the closest NG STAR type was the nonmosaic allele 18.001. ¶MIC 0.75 μg/mL was within susceptible range, although elevated compared with local reference strains. CLSI MIC cutoff for *N. gonorrhoeae* azithromycin susceptibility is MIC <1 μg/mL. However, CLSI has set a susceptible only interpretive breakpoint at this value, with the recommendation that this applies when azithromycin (1 g single dose) is used in combination with another antimicrobial, such as ceftriaxone. #Efflux pumps encoded in this strain include *mtrA*, *macA*, *macB*, and *farA*.

According to the multilocus sequence typing analysis at the Center of Genomic Epidemiology (https://www.genomicepidemiology.org) the sequence type was 7371 (Appendix Table 1, 2). The phylogenetic tree clustered our B196-JP22 isolate in a monophyletic terminal clade together with a urogenital strain (BioProject no. PRJNA560592; BioSample accession no. SAMN12591021) isolated in the city of Shenzhen, Guangdong Province, China, in January 2017 (*6*). Isolates in neighboring terminal clades were reported from Australia, Hong Kong, mainland China, and Vietnam (Appendix Figure).

This case demonstrates a triple failure: inadequate maternal screening for sexually transmitted infections and ineffective prophylaxis and treatment using fluoroquinolone-based ophthalmic agents against a multidrug-resistant *N. gonorrhoeae* strain. Robust maternal screening is critical to prevent perinatal transmission of sexually transmitted infections. US Centers for Disease Control and Prevention guidelines recommend repeat testing for *N. gonorrhoeae* and *C. trachomatis* during the second trimester for all pregnant women <25 years of age at increased risk (*7*). In contrast, prenatal care in Japan typically involves a single screening early in pregnancy, and routine screening for *N. gonorrhoeae* is uncommon (*8*).

In developed countries, the necessity of routine neonatal ocular prophylaxis is increasingly debated because it does not prevent *C. trachomatis* infection and the number of cases of gonococcal conjunctivitis is small (*9*). Several countries in Europe have discontinued prophylaxis without observing increased incidence of neonatal ophthalmia (*10*).

In Japan, fluoroquinolone-resistant *N. gonorrhoeae* strains are highly prevalent among adults; resistance rates are >80% for fluoroquinolones and ≈20% for macrolides (*2*). Genomic analysis of our isolate suggests further spread of resistant organisms in Asia, highlighting the importance of identifying this isolate in the region.

Given the low incidence of neonatal gonococcal conjunctivitis and the increasing prevalence of antimicrobial resistance in Japan, routine use of prophylactic ophthalmic solutions appears insufficient. Instead, systematic screening for maternal gonococcal infection, especially in late pregnancy, might be considered a more effective strategy to prevent vertical transmission.

Appendix Additional information about neonatal gonococcal conjunctivitis caused by fluoroquinolone-resistant *Neisseria gonorrhoeae*.
