# Supplementary material for: Neonatal Gonococcal Conjunctivitis Caused by Fluoroquinolone-Resistant Neisseria gonorrhoeae
Source: Emerg Infect Dis. 2025 Oct;31(10):2043–5. doi: 10.3201/eid3110.250895 (PMC12483108; doi:10.3201/eid3110.250895)
Supplement: Appendix — Additional information about neonatal gonococcal conjunctivitis caused by fluoroquinolone-resistant Neisseria gonorrhoeae. [file 25-0895-Techapp-s1.pdf]

Article DOI: https://doi.org/10.3201/eid3110.250895

EID cannot ensure accessibility for supplementary materials supplied by authors. Readers who have difficulty accessing supplementary content should contact the authors for assistance.

Neonatal Gonococcal Conjunctivitis Caused by Fluoroquinolone-Resistant *Neisseria gonorrhoeae*

Appendix

Patient findings on admission

Vital signs

Axillary temperature 37.0°C, respiratory rate 36 breaths/min, heart rate 133 bpm, and oxygen saturation 98% on room air.

Laboratory blood tests

Leukocyte counts of 10,740/µL, Platelet count of 380000/µL, Total bilirubin 5.4mg/dL, Creatinine 0.26mg/dL, Aspartate aminotransferase 32IU/L, Alanine aminotransferase 20IU/L, CRP of 0.13 mg/dL

Serologic tests for syphilis (RPR <0.4), *Treponema pallidum* antibody (<1.0), HIV (<1.0), hepatitis B surface antigen (<0.03 IU/mL), and hepatitis C antibody (<1.0) were all negative.

Urine tests

Protein (-), Glucose (-), Occult blood (2+), Nitrite (-), Leukocytes (-), Bacteria (-).

Methods

Neisseria gonorrhoeae B196-JP22 strain culture, genome DNA isolation, sequencing and analysis

The *Neisseria gonorrhoeae* isolated from the patient (designated B196-JP22) was cultured in chocolate blood agar at 37°C in a 5% CO_2 humid environment incubator. After DNA extraction with the Nucleospin Microbial DNA kit, we prepared a genome library with the Oxford Nanopore Technologies Native Bar Coding kit and sequenced it with a FLO-MIN114 v.R10 flow cell set on the MinION Mk1C device. Sequences for MLST typing were confirmed by Sanger sequencing. Genome analysis was performed using tools from: MLSTVERSE (*1*) to obtain the fastq file, BV-BRC (*2*) to assemble the draft genome contig fasta file, Proksee (*3*) to annotate the genome, Pathogenwatch (https://pathogen.watch), pubMLST (*4*), NCBI, BV-BRC databases to gather previously reported *Neisseria gonorrhoeae* strain genomes and to generate the phylogenetic tree and the SNP matrix analysis, ResFinder4.7.2 (*5,6*) and AMRprofiler (*7*) to analyze antimicrobial resistance genes.

Molecular characterization of the *Neisseria gonorrhoeae* B196-JP22 strain

The draft whole genome sequence was 2234371bp long encompassing 3 contigs: the smallest contig had 3529bp and the largest, contig1, had 2222712bp covering the whole chromosome. The quality and contiguity of the assembly was reflected in the N50: 2222712 value. The GC content was 52.3%. Genome blast comparison with the WHO 2024 Reference strain Alpha (*8*), revealed the presence of a 4143bp cryptic plasmid (NZ_CP145058.1) in contig3.

MLST typing

Initial uploading of B196-JP22 draft whole genome sequence onto the MLST-2.0 typing platform at the CGE revealed that the closet ST type was 7371. However, the adk, aroE and

pdhC loci did not share 100% identity with the adk_39, aroE_170 and pdhC_153 alleles corresponding to the ST7371. Thus, we amplified these loci by PCR and performed Sanger sequencing to verify the sequences using specific primers (Appendix Table 1). The obtained sequences were uploaded onto CGE and the allele types matched 100% with those of the ST7371 (Appendix Table 2).

Genetic analysis of antibiotic susceptibility

The B196-JP22 genome contained SNPs and acquired genes mediating antimicrobial resistance (*9,10*) consistent with the antibiotic susceptibility test results (Table, https://wwwnc.cdc.gov/EID/article/31/10/25-0895-T1.htm). Apart from that, the B196-JP22 genome contained sulfonamide and tetracycline resistance determinant SNPs: an arginine to serine substitution at position 228 in the protein encoded by the folP gene (folP_R228S) and a valine to methionine substitution at position 57 in the protein encoded by rpsJ (rpsJ_V57M) gene respectively (*11*).

B196-JP22 lineage relation to worldwide *Neisseria gonorrhoeae* strains

In an effort to trace the origin and spread of B196-JP22 we performed a phylogenetic tree analysis on Pathogenwatch (Appendix Figure). We input our B196-JP22 isolate and 1368 worldwide *Neisseria gonorrhoeae* including strains that out of the 7 house-keeping genes used in MLST typing, had at least 4 loci sharing 100% identity with B196-JP22 (MLST types: ST7371, ST1583, ST1921, ST1922, ST 8109, ST12078, ST12242, ST12243, ST14421, ST14591); 2024 WHO *Neisseria gonorrhoeae* reference strains (*8*) and publicly available Japanese strains (regardless of MLST ST type). The analysis showed that B196-JP22 had a direct shared ancestry with the SAMN12591021 People's Republic of China strain, however it was allocated in a long branch. The SNPs matrix revealed 1175 differences between these 2 strains. Interestingly, B196-

JP22 was closer related to strains reported in Australia (*13*), Hong Kong Special Administrative Region, People's Republic of China (*14*), Vietnam (*15*) and the U.S (*16,17*), than to strains reported in Japan (*18,19*). Future genome sequencing applied to the majority of *Neisseria gonorrhoeae* strains collected in Japan may help assess whether B196-JP22 is a new strain in our country.

References

1. Matsumoto Y, Kinjo T, Motooka D, Nabeya D, Jung N, Uechi K, et al. Comprehensive subspecies identification of 175 nontuberculous mycobacteria species based on 7547 genomic profiles. Emerg Microbes Infect. 2019;8:1043–53. PubMed https://doi.org/10.1080/22221751.2019.1637702

2. Olson RD, Assaf R, Brettin T, Conrad N, Cucinell C, Davis JJ, et al. Introducing the Bacterial and Viral Bioinformatics Resource Center (BV-BRC): a resource combining PATRIC, IRD and ViPR. Nucleic Acids Res. 2023;51(D1):D678–89. PubMed https://doi.org/10.1093/nar/gkac1003

3. Grant JR, Enns E, Marinier E, Mandal A, Herman EK, Chen CY, et al. Proksee: in-depth characterization and visualization of bacterial genomes. Nucleic Acids Res. 2023;51(W1):W484–92. PubMed https://doi.org/10.1093/nar/gkad326

4. Jolley KA, Bray JE, Maiden MCJ. Open-access bacterial population genomics: BIGSdb software, the PubMLST.org website and their applications. Wellcome Open Res. 2018;3:124. PubMed https://doi.org/10.12688/wellcomeopenres.14826.1

5. Bortolaia V, Kaas RS, Ruppe E, Roberts MC, Schwarz S, Cattoir V, et al. ResFinder 4.0 for predictions of phenotypes from genotypes. J Antimicrob Chemother. 2020;75:3491–500. PubMed https://doi.org/10.1093/jac/dkaa345

6. Camacho C, Coulouris G, Avagyan V, Ma N, Papadopoulos J, Bealer K, et al. BLAST+: architecture and applications. BMC Bioinformatics. 2009;10:421. PubMed https://doi.org/10.1186/1471-2105-10-421

7. Skoulakis A, Kostoula V, Daniilidis K, Gkountinoudis CG, Petinaki E, Hatzigeorgiou AG. AmrProfiler: a comprehensive tool for identifying antimicrobial resistance genes and mutations across species. Nucleic Acids Res. 2025;53(W1):W20–31. PubMed https://doi.org/10.1093/nar/gkaf400

8. Unemo M, Sánchez-Busó L, Golparian D, Jacobsson S, Shimuta K, Lan PT, et al. The novel 2024 WHO *Neisseria gonorrhoeae* reference strains for global quality assurance of laboratory investigations and superseded WHO *N. gonorrhoeae* reference strains-phenotypic, genetic and reference genome characterization. J Antimicrob Chemother. 2024;79:1885–99. PubMed https://doi.org/10.1093/jac/dkae176

9. Warner DM, Shafer WM, Jerse AE. Clinically relevant mutations that cause derepression of the *Neisseria gonorrhoeae* MtrC-MtrD-MtrE Efflux pump system confer different levels of antimicrobial resistance and in vivo fitness. Mol Microbiol. 2008;70:462–78. PubMed https://doi.org/10.1111/j.1365-2958.2008.06424.x

10. Unemo M, Shafer WM. Antimicrobial resistance in *Neisseria gonorrhoeae* in the 21st century: past, evolution, and future. Clin Microbiol Rev. 2014;27:587–613. PubMed https://doi.org/10.1128/CMR.00010-14

11. Rice PA, Shafer WM, Ram S, Jerse AE. *Neisseria gonorrhoeae:* drug resistance, mouse models, and vaccine development. Annu Rev Microbiol. 2017;71:665–86. PubMed https://doi.org/10.1146/annurev-micro-090816-093530

12. Argimón S, Abudahab K, Goater RJE, Fedosejev A, Bhai J, Glasner C, et al. Microreact: visualizing and sharing data for genomic epidemiology and phylogeography. Microb Genom. 2016;2:e000093. PubMed https://doi.org/10.1099/mgen.0.000093

13. Williamson DA, Chow EPF, Gorrie CL, Seemann T, Ingle DJ, Higgins N, et al. Bridging of *Neisseria gonorrhoeae* lineages across sexual networks in the HIV pre-exposure prophylaxis era. Nat Commun. 2019;10:3988. PubMed https://doi.org/10.1038/s41467-019-12053-4

14. Bristow CC, Mortimer TD, Morris S, Grad YH, Soge OO, Wakatake E, et al. Whole-genome sequencing to predict antimicrobial susceptibility profiles in *Neisseria gonorrhoeae*. J Infect Dis. 2023;227:917–25. PubMed https://doi.org/10.1093/infdis/jiad027

15. Lan PT, Golparian D, Ringlander J, Van Hung L, Van Thuong N, Unemo M. Genomic analysis and antimicrobial resistance of *Neisseria gonorrhoeae* isolates from Vietnam in 2011 and 2015–16. J Antimicrob Chemother. 2020;75:1432–8. PubMed https://doi.org/10.1093/jac/dkaa040

16. Grad YH, Harris SR, Kirkcaldy RD, Green AG, Marks DS, Bentley SD, et al. Genomic epidemiology of gonococcal resistance to extended-spectrum cephalosporins, macrolides, and fluoroquinolones in the United States, 2000–2013. J Infect Dis. 2016;214:1579–87. PubMed https://doi.org/10.1093/infdis/jiw420

17. Gernert KM, Seby S, Schmerer MW, Thomas JC IV, Pham CD, Cyr SS, et al.; Antimicrobial-Resistant *Neisseria gonorrhoeae* Working Group. Azithromycin susceptibility of *Neisseria gonorrhoeae* in the USA in 2017: a genomic analysis of surveillance data. Lancet Microbe. 2020;1:e154–64. PubMed https://doi.org/10.1016/S2666-5247(20)30059-8

18. Yahara K, Nakayama SI, Shimuta K, Lee KI, Morita M, Kawahata T, et al. Genomic surveillance of *Neisseria gonorrhoeae* to investigate the distribution and evolution of antimicrobial-resistance determinants and lineages. Microb Genom. 2018;4:e000205. PubMed

19. Sánchez-Busó L, Golparian D, Corander J, Grad YH, Ohnishi M, Flemming R, et al. The impact of antimicrobials on gonococcal evolution. Nat Microbiol. 2019;4:1941–50. PubMed https://doi.org/10.1038/s41564-019-0501-y

Appendix Table 1. PCR primers for amplification and sequencing of house-keeping genes

Primer type	Primer
Amplification primer	
adk-P1B *	5-CCAAGCCGTGTAGAATCGTAAACC-3
adk-P2B *	5-TGCCCAATGCGCCCAATAC-3
aroE-P1B *	5-TTTGAAACAGGCGGTTGCGG-3
aroE-P2B *	5-CAGCGGTAATCCAGTGCGAC-3
pdhC-B196F †	5-GGCATTCGGTTTTCAGACGG-3
pdhC-B196R †	5-TTCCAACGTATCGGCGACTT-3
Sequencing primers	
adk-S1A *	5′-AGGCWGGCACGCCCTTGG-3′
adk-S2 *	5′-CAATACTTCGGCTTTCACGG-3′
aroE-S1A *	5-GCGGTCAAYACGCTGRTK-3
pdhC-S2 *	5′-ATCGGCTTTGATGCCGTATTT-3′

* Primers designed by Birtles A. are published on the pubMLST Web site.

† Primers designed by the authors.

Appendix Table 2. B196-JP22 MLST allele types analyzed on MLST-2.0

Locus	Identity %	Coverage %	Alignment Length	Allele Length	Gaps	Allele
abcZ	100	100	433	433	0	abcZ_59
adk	100	100	465	465	0	adk_39
aroE	100	100	490	490	0	aroE_170
fumC	100	100	465	465	0	fumC_111
gdh	100	100	501	501	0	gdh_148
pdhC	100	100	480	480	0	pdhC_153
pgm	100	100	450	450	0	pgm_65

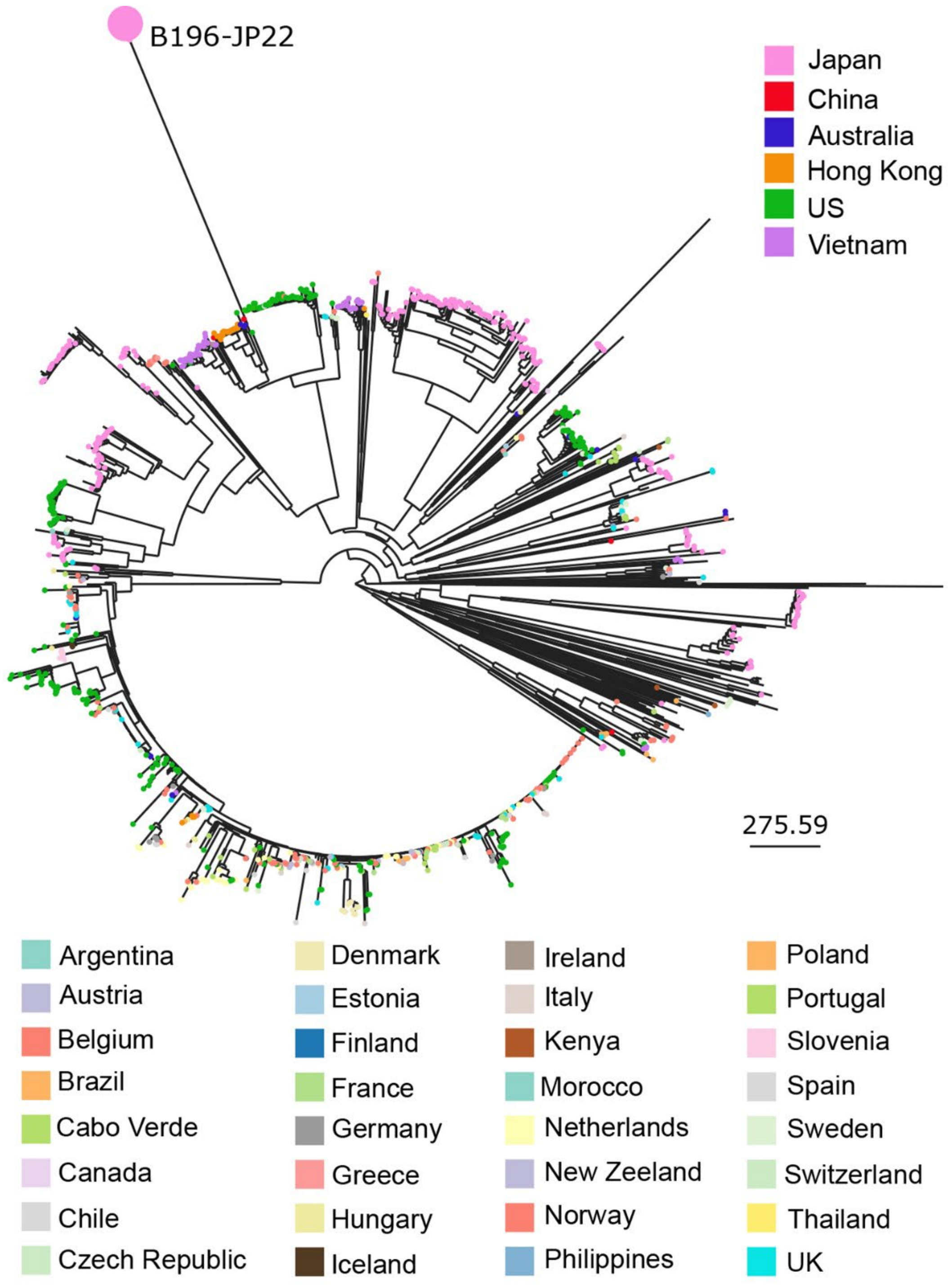

Appendix Figure. Phylogenetic tree. Above are color keys of countries where strains closely related to B196-JP22 were isolated. Below are color keys of countries where the rest of the strains where isolated. Scale bar represents branch length corresponding to the number of SNP differences. Visualization of strain country origin was performed using Microreact (*12*).
